# Tolerance to stress conditions associated with food safety in *Campylobacter jejuni* strains isolated from retail raw chicken

**DOI:** 10.1038/s41598-019-48373-0

**Published:** 2019-08-15

**Authors:** Euna Oh, Katelyn J. Andrews, Lynn M. McMullen, Byeonghwa Jeon

**Affiliations:** 1 School of Public Health, University of Alberta, Edmonton, Alberta Canada; 2 Department of Agricultural, Food and Nutritional Science, University of Alberta, Edmonton, AB Canada; 3 Present Address: Environmental Health Sciences, School of Public Health, University of Minnesota, St. Paul, MN USA

**Keywords:** Bacteriology, Applied microbiology

## Abstract

*Campylobacter jejuni* is a microaerophilic foodborne pathogen that is sensitive to stress conditions. However, it is not yet understood how this stress-sensitive pathogen may cause a significant number of cases of human gastroenteritis worldwide. In this study, we examined stress tolerance in 70 *C*. *jejuni* strains isolated from retail chicken under several stress conditions related to food safety. Compared to oxygen-sensitive (OS) strains of *C*. *jejuni*, *C*. *jejuni* strains with increased aerotolerance, such as hyper-aerotolerant (HAT) and aerotolerant (AT) strains, were more tolerant to peracetic acid, refrigeration and freeze-thaw stresses. However, the levels of thermotolerance and hyper-osmotolerance were not associated with the aerotolerance level of *C*. *jejuni*. The HAT and AT strains of *C*. *jejuni* exhibited significantly increased activities of catalase and superoxide dismutase (SOD), compared to the OS strains. Consistently, the HAT and AT strains were highly tolerant to oxidants, such as hydrogen peroxide, cumene hydroperoxide and menadione, compared to the OS strains. The AT and HAT strains that were tolerant to stresses, particularly peracetic acid and refrigeration, predominantly belonged to multilocus sequence typing (MLST) clonal complex (CC)-21. This study shows that oxidative stress resistance plays a role in determining the differential level of aerotolerance in *C*. *jejuni* and that AT and HAT strains of *C*. *jejuni* are more tolerant to oxidants and low temperatures than OS strains.

## Introduction

*Campylobacter* spp. is one of the primary bacterial causes of gastroenteritis worldwide^1^. Since campylobacters are isolated from a range of food-producing and companion animals, wildlife, and environmental sources, *Campylobacter* outbreaks are caused by various sources, such as water, raw milk, and even mud^2,3^. However, the consumption of contaminated poultry meat is the primary cause of human campylobacteriosis^2^. Although a number of poultry species carry pathogenic species of *Campylobacter* (i.e., *Campylobacter jejuni* and *Campylobacter coli*)^4^, chickens are most frequently implicated in human infections^5^. In the EU, for example, it has been estimated that 50–80% of human campylobacteriosis cases are attributed to chicken^5^. Consistently, studies show that retail chicken meats are frequently contaminated with *Campylobacter*. Approximately 61% of retail chicken in the UK is contaminated with *Campylobacter*^6^, and 62% of raw chicken legs in Canada^7^. Due to the high frequency of *Campylobacter* contamination of chicken, it has been estimated that a two-log reduction of *Campylobacter* counts on chicken carcasses would decrease the chances of human infection with *Campylobacter* by 30-fold^8^. To decrease *Campylobacter* contamination of poultry carcasses, various intervention methods have been employed in poultry processing, including physical treatment with hot water and steam, chilling and freezing of carcasses, and chemical decontamination^9^. Among the antimicrobial agents available for poultry carcasses^10^, peroxyacetic acid (PAA) is widely used by the poultry industry in many countries because the antimicrobial activity of PAA is highly effective^11^, and it decomposes to acetic acid, oxygen, and water without causing toxicity and environmental risks^12^. Given that multiple methods used to reduce *Campylobacter* loads on poultry carcasses, *Campylobacter* should overcome these stress conditions prior to the initiation of foodborne infection in humans.

Since stress tolerance contributes to bacterial survival under harsh environmental conditions, the stress tolerance of foodborne pathogens plays a significant role in food safety^13^. Compared to most other enteric pathogens (e.g. *Salmonella* and enterohemorrhagic *Escherichia coli*) that efficiently adapt to and survive under stress conditions^14^, *C*. *jejuni* is known to be highly sensitive to environmental stress primarily due to the lack of several stress tolerance genes that are commonly found in other enteric pathogenic bacteria. For instance, *C*. *jejuni* is microaerophilic and sensitive to atmospheric oxygen, whereas *E*. *coli* and *Salmonella* grow both aerobically and anaerobically. Additionally, *C*. *jejuni* lacks cold stress proteins^15^ and RpoS^16,17^, a key stress response regulator and in *E*. *coli* and *Salmonella*^18,19^. It is not clearly understood how *C*. *jejuni* may survive under stress conditions present in food processing, transportation, preservation, and cooking, and is increasingly responsible for gastroenteritis despite its less-conserved stress tolerance mechanisms compared to other foodborne pathogens. Our recent studies showed that certain strains of *Campylobacter*, both *C*. *jejuni* and *Campylobacter coli*, are highly tolerant to aerobic stress and that such aerotolerant *Campylobacter* is highly prevalent on retail raw chicken^20,21^. Interestingly, *C*. *coli* strains are more aerotolerant than *C*. *jejuni* strains^21^. Furthermore, clinical strains of *C*. *jejuni* with tolerance to environmental stress form genetically unique clusters and are more frequently involved in human infections in Canada compared to oxygen-sensitive *C*. *jejuni*^22^. Although the previous study performed an extensive analysis using 121 clinical strains of *C*. *jejuni*, the stress tolerance of *C*. *jejuni* strains isolated from retail poultry has not been investigated although retail chicken meat is the major reservoir transmitting *C*. *jejuni* to humans. To fill this important knowledge gap, we examined the tolerance of *C*. *jejuni* strains isolated from retail chicken meats to five different stress conditions (i.e., disinfectant, refrigeration, freeze-thaw, heat, and high salt concentrations), which *C*. *jejuni* may encounter during foodborne transmission to humans.

## Results

### Disinfectant resistance in *C*. *jejuni* isolates from retail chicken

**Figure 1:**
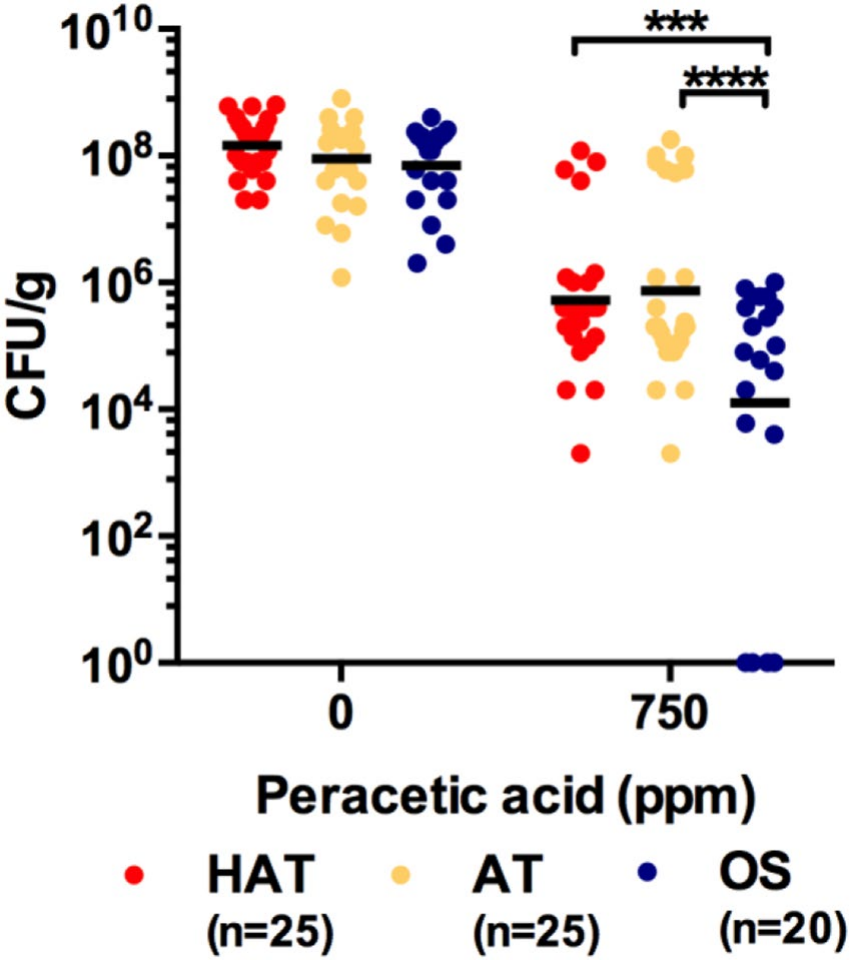
Tolerance to peracetic acid (PAA) in 70 *C*. *jejuni* isolates from retail chicken. Solid black bar indicates the mean CFU. The experiment was repeated three times and produced similar results. Two-way ANOVA was used to compare the results in the different aerotolerance groups. ***P < 0.005, ****P < 0.0001.

Since chicken carcasses are usually treated with disinfectants during processing, we measured the tolerance of 70 *C*. *jejuni* strains to PAA, a common disinfectant used by the poultry industry. The PAA exposure significantly reduced the CFU levels of *C*. *jejuni* strains. More significant reduction (ca. 3.7 log CFU/g) was observed in the OS strains compared to the AT and HAT strains (ca. 2.1 and 2.5 log CFU/g, respectively) (Fig. 1). Although most of the tested strains of *C*. *jejuni* exhibited a certain level of PAA tolerance, the results showed that AT and HAT strains of *C*. *jejuni* were more tolerant to PAA than OS strains.

### Increased capability of ROS detoxification in AT and HAT strains of *C*. *jejuni*

**Figure 2:**
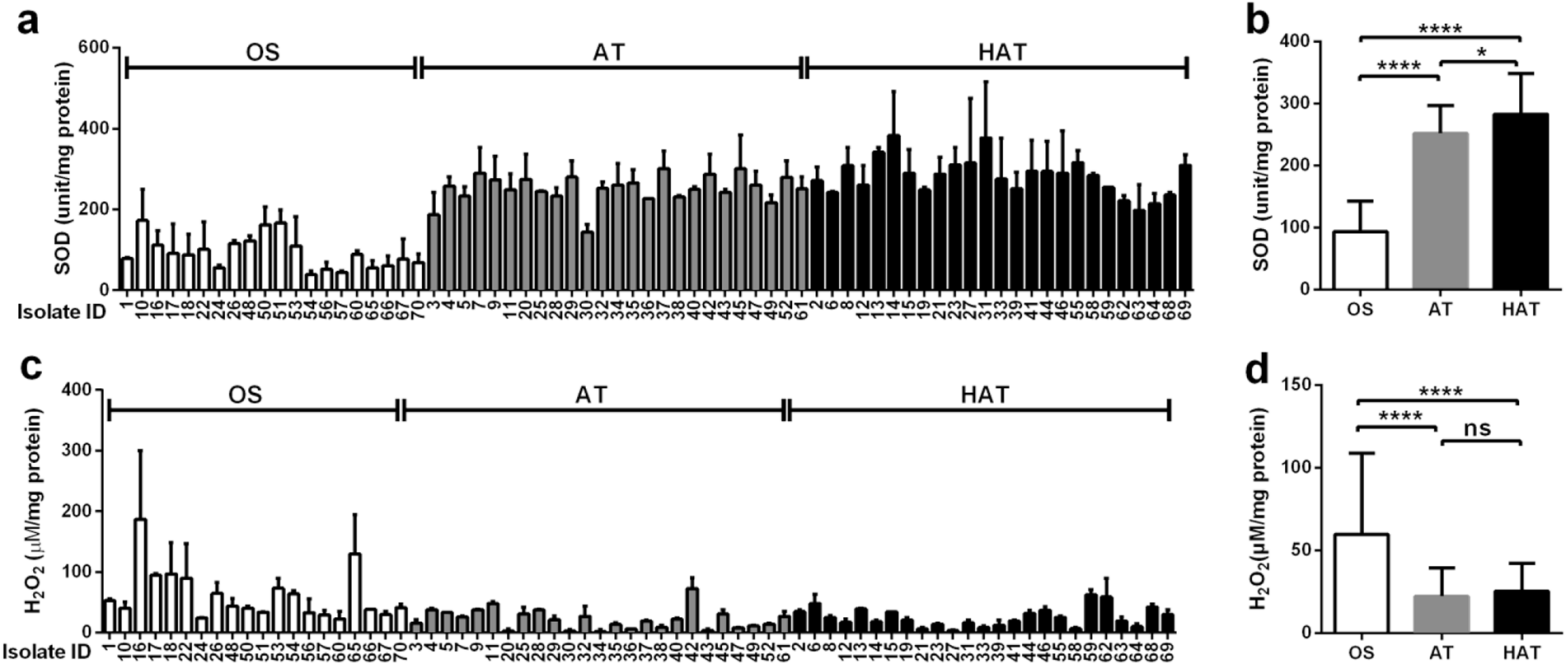
Superoxide dismutase (SOD) and catalase activities in *C*. *jejuni* isolates from retail chicken. SOD activities in 70 strains of *C*. *jejuni* (**a**) and the average of SOD activities in OS, AT, and HAT strains (**b**). Catalase activities in 70 strains of *C*. *jejuni* (**c**) and the average of catalase activities in OS, AT, and HAT strains (**d**). The results show the means and standard deviations of a single experiment with triplicate samples, and the experiment was repeated three times. Two-way ANOVA was used for statistical analysis. ns: not significant, *P < 0.05, ****P < 0.0001.

Since oxidative stress defense plays an important role in aerotolerance by detoxifying ROS^23,24^ and AT and HAT strains of *C*. *jejuni* were highly resistant to PAA (Fig. 1), an organic peroxide, we hypothesized that AT and HAT strains of *C*. *jejuni* might be more capable of detoxifying ROS than OS strains. To examine this hypothesis, we measured the activities of superoxide dismutase (SOD) and catalase, two important oxidative stress defense enzymes detoxifying the superoxide anion and H_2_O_2_, respectively. Interestingly, the SOD activity was significantly higher in the AT and HAT strains than the OS strains (Fig. 2a,b). The catalase activity was determined by measuring the intracellular level of H_2_O_2_ in *C*. *jejuni*; thus, a lower H_2_O_2_ level indicates a higher catalase activity. The AT and HAT strains of *C*. *jejuni* accumulated less H_2_O_2_ than the OS strains (Fig. 2c,d), suggesting that the catalase activity is higher in the AT and HAT strains than the OS strains. These findings clearly demonstrated that AT and HAT strains of *C*. *jejuni* are more resistant to oxidative stress than OS strains; this may contribute to the enhanced PAA tolerance of AT and HAT strains.

**Figure 3:**
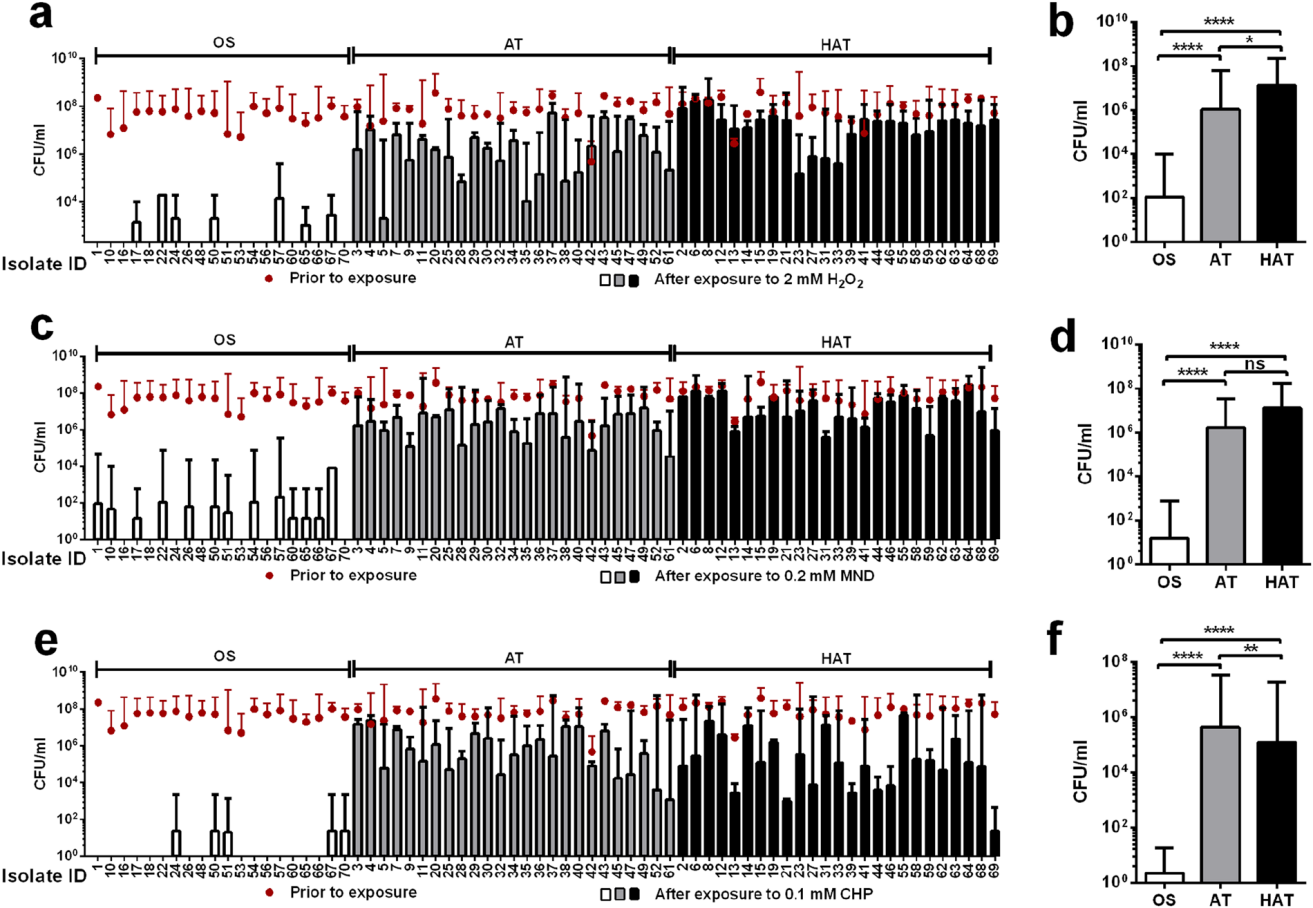
Tolerance to oxidants in 70 strains of *C*. *jejuni* from retail chicken. Viability after 1 h exposure to hydrogen peroxide (H_2_O_2_) in 70 strains (**a**) and the means of CFU in OS, AT, and HAT strains (**b**), menadione (MND) in 70 strains (**c**) and the means of CFU in OS, AT, and HAT strains (**d**), and cumene hydroperoxide (CHP) in 70 strains (**e**) and the means of CFU in OS, AT, and HAT strains (**f**). The results show the means and standard deviations of the viability (CFU/ml) in a single experiment with triplicate samples. The CFU level of the inoculum is indicated with a dot graph in red. The experiment was repeated three times. Statistical significance was carried out with two-way ANOVA. ns: not significant, *P < 0.05, **P < 0.01, ****P < 0.0001.

The 70 strains of *C*. *jejuni* were exposed to three different oxidants, including H_2_O_2_, cumene hydroperoxide (CHP; an organic peroxide), and menadione (MND; a superoxide generator). Despite variations depending on the strain and the oxidant (Fig. 3a,c,e), the viability reduction was significant in the OS strains compared to the AT and HAT strains (Fig. 3b,d,f). The results of the oxidative stress defense enzyme assays (Fig. 2) and the susceptibility tests (Fig. 3) consistently showed that HAT and AT strains of *C*. *jejuni* were highly tolerant to oxidative stress.

### Tolerance to refrigeration, freeze-thaw, and heat in *C*. *jejuni* strains from retail chicken

**Figure 4:**
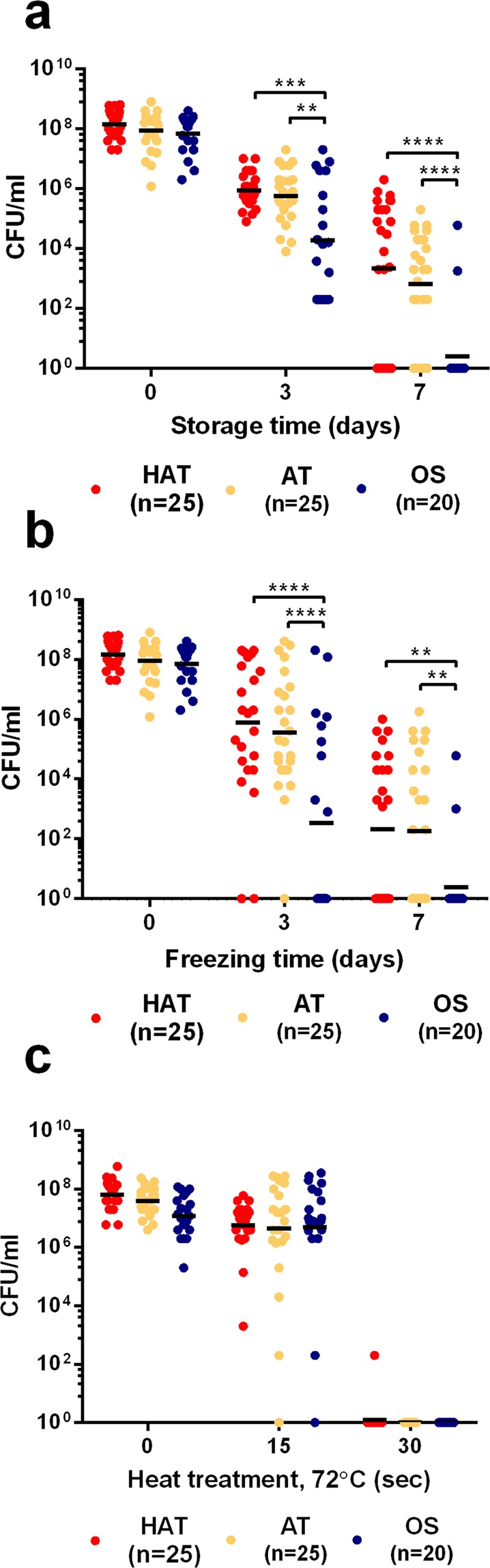
Tolerance to refrigeration (**a**), freezing (**b**), and heat (**c**) stresses in 70 strains of *C*. *jejuni* from retail chicken. The results are representative of three independent experiments. Similar results were observed in all three experiments. Solid black bars show the mean CFU. Statistical significance was determined using two-way ANOVA. **P < 0.01, ***P < 0.005, ****P < 0.0001.

After packaging at processing plants, chicken carcasses are refrigerated or frozen for distribution and preservation. Thus, refrigeration and freezing are harsh stress conditions that *C*. *jejuni* encounters in the chicken supply system. In addition, *C*. *jejuni* will be exposed to high temperatures during cooking. The OS strains exhibited a significant CFU reduction at a refrigeration temperature. Storing at 4 °C for seven days reduced bacterial counts by approximately 4.9, 5.1, and 7.4 CFU/ml in the HAT, AT, and OS strains of *C*. *jejuni*, respectively (Fig. 4a). Similarly, the OS strains of *C*. *jejuni* were more sensitive to freeze-thaw stress that the AT and HAT strains. More than half (ca. 52%) of HAT and AT strains survived at −20 °C for seven days; however, only two OS strains were detected after seven days (Fig. 4b). Unlike the results of the refrigeration and freeze-thaw tolerance tests, the aerotolerance level was not associated with thermotolerance as only one HAT strain and one AT strain survived after exposure to 70 °C for 30 sec (Fig. 4c). Based on the findings, *C*. *jejuni* strains from retail chicken exhibited different levels of tolerance to cold and heat stresses, and AT and HAT *C*. *jejuni* were highly tolerant to refrigeration and freeze-thaw stresses, but not to heat stress.

### Tolerance to hyperosmotic stress

**Figure 5:**
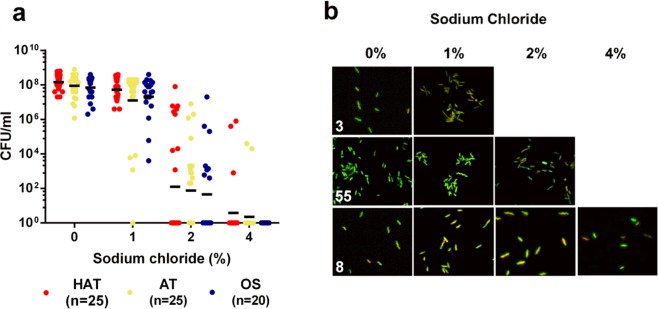
Osmotolerance in 70 strains of *C*. *jejuni* from retail chicken and morphological changes under osmotic stress. (**a**) The viability of 70 strains of *C*. *jejuni* under different NaCl concentrations (1, 2, and 4%). Solid horizontal lines indicate the mean values. (**b**) Morphological changes in *C*. *jejuni* strains under osmotic stress. *C*. *jejuni* was stained with SYTO9 and propidium iodine, and the strain numbers are indicated in the figure. The experiments were repeated three times. Two-way ANOVA was used for statistical analysis.

Hyperosmotic stress is a stress condition for *Campylobacter* in food preservation and cooking, such as marination. When exposed to different concentrations of NaCl (1%, 2%, and 4%), all of the tested strains except for one AT strain survived at 1% NaCl, and approximately 36% of HAT, 48% of AT, and 40% of OS strains survived at 2% NaCl with a wide range of CFU levels (Fig. 5a). Whereas five AT and HAT strains survived at 4% NaCl, none of the OS strains survived; however, the difference was not statistically significant (Fig. 5a). Based on the results of fluorescence microscopic analysis, *C*. *jejuni* exhibited heterogeneous morphology with mixed populations of helical rod, elongated, and coccoid cells depending on the strain (Fig. 5b).

### MLST sequence types of stress-tolerant *C*. *jejuni* strains

**Figure 6:**
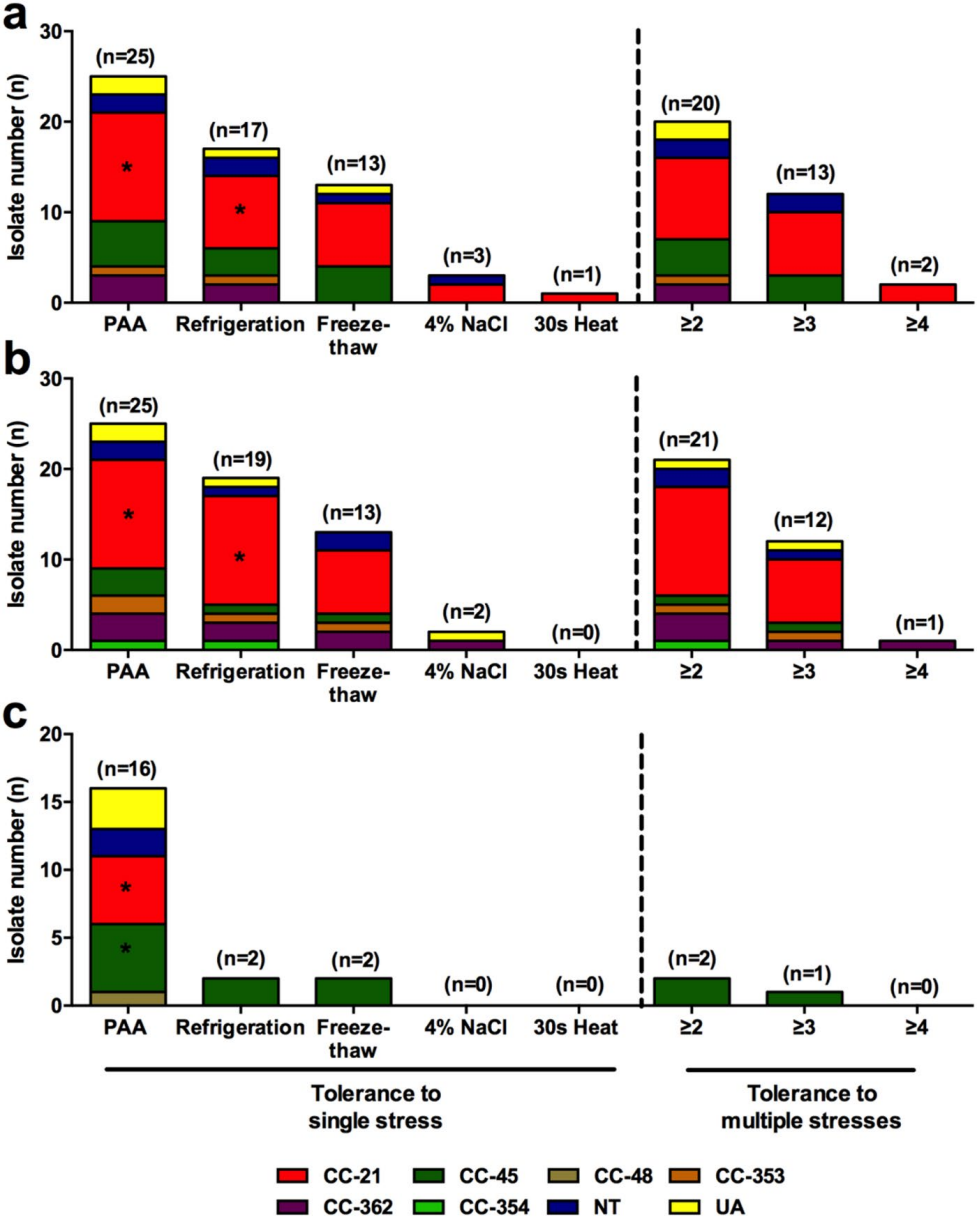
MLST clonal complexes of HAT (**a**), AT (**b**), and OS (**c**) strains of *C*. *jejuni* tolerant to stress conditions. Statistical significance was determined using two-way ANOVA. *P < 0.01.

The MLST sequence types of the 70 strains of *C*. *jejuni* from raw chicken have been reported in our previous study^20^. CC-21 was predominant in AT and HAT strains that were tolerant to stress conditions, particularly PAA and refrigeration (Fig. 6a,b). Among the OS strains tolerant to PAA, CCs-21 and 45 were dominant with a statistical significance (Fig. 6c). Commonly, CC-21 is the primary MLST sequence type in stress-tolerant *C*. *jejuni* strains from raw chicken.

## Discussion

Most foodborne pathogenic bacteria of public health concern usually originate from animals^25^. Since poultry is the major reservoir for *Campylobacter*, poultry carcasses are likely to be contaminated by *Campylobacter* during processing, particularly defeathering and evisceration^26^. To maintain food quality by reducing spoilage and pathogenic bacteria on poultry carcasses, various intervention methods are used by the poultry industry, including low storage temperature, marination, modified atmospheric gas packaging, and antimicrobial disinfectants^27^.

PAA is a disinfectant widely used to decontaminate poultry carcasses in process water for washing, rinsing, and chilling due to its strong antimicrobial efficacy^28,29^. Interestingly, AT and HAT strains of *C*. *jejuni* exhibited enhanced tolerance to PAA compared to OS strains (Fig. 1). This may be attributed to the increased oxidative stress defense in AT and HAT strains (Fig. 2) as PAA is a mixture of H_2_O_2_ and acetic acid. Whereas most other bacteria harbor redundant copies of genes encoding ROS-detoxification enzymes, such as KatA, SodB and AhpC, *C*. *jejuni* possesses only a single gene copy encoding the enzymes. Although AhpC is the major enzyme contributing to aerotolerance in *C*. *jejuni*^23^, the other two enzymes (i.e., KatA and SodB) also affect the viability of *C*. *jejuni* under aerobic conditions^24^. Presumably, the increased aerotolerance would be associated with the augmented activities of oxidtative stress defense enzymes, which also may increase the capability of decomposing PAA. This is also supported by the augmented survivality of AT and HAT strains after exposure to various types of oxidants (Fig. 3).

Interestingly, AT and HAT strains of *C*. *jejuni* were more tolerant to refrigeration and freezing temperatures compared to OS strains (Fig. 4a,b). Most bacterial species, such as *E*. *coli*, *Salmonella*, and *Bacillus*, produce cold shock proteins upon a temperature downshift^30–32^. However, *C*. *jejuni* does not possess genes encoding cold shock proteins^15^, which suggests that *C*. *jejuni* may have other tolerance mechanisms to respond to cold shocks. Studies thus far have shown oxidative stress defense, particularly SodB, plays an important role in the cold stress tolerance of *Campylobacter*. A knockout mutation of *sodB* increases the sensitivity of *C*. *jejuni* to both superoxide and peroxide stress^33,34^. The level of *sodB* expression in *C*. *jejuni* increases by exposure to cold-shock^35^, and a *sodB* mutation makes *Campylobacter* more susceptible to freeze-thaw stress than the wild type^36,37^. However, the survival of a *sodB* mutant is comparable to that of the wild type in the absence of oxygen^36^, indicating that oxidative stress impacts *C*. *jejuni*’s ability to survive under freeze-thaw conditions. Notably, the SOD activities were determined to be significantly higher in AT and HAT strains than OS strains (Fig. 2a,b), and the AT and HAT strains were more tolerant to MND, a superoxide generator (Fig. 3c,d). Presumably, the elevated levels of SOD activity may possibly contribute to the enhanced survival of AT and HAT strains under refrigeration and freeze-thaw stress conditions. The association of cold stress with oxidative stress defense has also been reported in some other bacteria. For instance, exposure of *Pseudomonas fluorescens* MTCC 667, an isolate from Antarctic soil, to low temperature (4 °C) increases the production of ROS and elevates the activity of SOD^38^. Although molecular mechanisms still remain to be explained, our findings and the studies done by others consistently suggest oxidative stress defense may affect *C*. *jejuni* tolerance to cold and freezing stresses. *C*. *jejuni* strains from retail chicken were relatively sensitive to heat stress (Fig. 3c), compared to human clinical strains of *C*. *jejuni* in our previous study^22^. The reason for the different levels of thermotolerance between chicken isolates and clinical isolates of *C*. *jejuni* remains unknown.

*C*. *jejuni* is sensitive to hyperosmotic stress^39^ and is easily inactivated at >2% NaCl^40^. Thus, high (1.5% ~ 3%) salt concentrations in marinated poultry meat^41^ would be another stress to *C*. *jejuni* during foodborne transmission. NaCl is a general food preservative and inhibits the growth of foodborne pathogens in foods^42^. Cameron *et al*. reported that exposure to 1% NaCl modestly upregulates oxidative stress genes, such as *katA* and *sodB*, in *C*. *jejuni*, suggesting oxidative stress defense may affect osmotic stress response^43^. However, in the current study, the level of hyper-osmotolerance was not related to aerotolerance, although the results showed the level of hyper-osmotolerance is highly variable depending on the strain (Fig. 5). In *C*. *jejuni*, capsular polysaccharides (CPS) are involved in hyper-osmotolerance as mutations in the genes involved in genes encoding CPS, such as *kpsM*, *kpsS*, and *kpsC*, significantly (ca. 100-fold) increase *C*. *jejuni* susceptibility to 1% NaCl^43^. To maintain the intracellular turgor pressure properly under hyper-osmotic stress, bacteria generally accumulate solutes by increasing the uptake of K^+^ and synthesizing osmolytes, such as trehalose and glutamate^44^. Although molecular mechanisms for osmotolerance have not yet been elucidated in *C*. *jejuni*, it has been reported that highly-frequent spontaneous variations in housekeeping genes related to purine biosynthesis, such as *purF* and *apt*, is associated with *C*. *jejuni* response to hyperosmotic stress^45^.

This study demonstrated that *C*. *jejuni* strains isolated from retail raw chicken were tolerant to several different stress conditions that may affect the survival of *C*. *jejuni* in chicken and that aerotolerance is significantly related to *C*. *jejuni* tolerance to PAA and refrigeration and freezing temperatures. Further comparative genomics analysis as follow-up studies will help us elucidate the molecular mechanisms underlying stress tolerance in *C*. *jejuni*.

## Methods

### Bacterial strains and culture conditions

Seventy strains of *C*. *jejuni* were isolated from retail chicken meat in our previous study^29^. The strains were routinely grown on Muller-Hinton (MH) media at 42 °C under microaerobic condition (5% O_2_, 10% CO_2_, 85% N_2_).

### Stress tolerance testing of *C*. *jejuni*

Stress tolerance testing was performed as described in our previous study with slight modifications^22^. *C*. *jejuni* strains were grown on MH agar at 42 °C for overnight under microaerobic condition. The strains were harvested in MH broth and resuspended in fresh MH broth to an optical density at 600 nm (OD600) of 0.1 prior to testing.

1. **Tolerance to PAA:** Raw chicken skin (0.3 g) was prepared with a sterilized razor, and each piece of chicken skin was inoculated *C*. *jejuni* suspension (approximately 10^8^ CFU). The chicken skin spiked with *C*. *jejuni* suspension was incubated at 4 °C for 1 h under microaerobic conditions and dipped in 750 ppm PAA solution for 15 sec and immediately washed in ultra-pure water. The chicken skin was transferred into a 15 ml tube with 2 ml fresh MH broth and vortexed for 2 min. Bacterial count was determined with a serial dilution and cultivation on Preston *Campylobacter*-selective agar. The experiment was repeated at least three times.
2. **Tolerance to refrigeration and freeze-thaw:** *C*. *jejuni* suspensions were placed into a 96-well plate and incubated at 4 °C for 3 and 7 days for refrigeration stress and also incubated at −20 °C for 3 and 7 days for freeze-thaw stress. After 3 and 7 days, the incubated *C*. *jejuni* suspensions were serially diluted and spread on MH agar for enumeration.
3. **Osmotolerance test:** The suspension of *C*. *jejuni* was diluted 10 times and spotted on MH agar supplemented with 1, 2 and 4% sodium chloride. The culture plates were incubated at 42 °C overnight.

### Catalase and SOD activity test

*C*. *jejuni* strains were inoculated on MH agar and harvested with 1X PBS buffer (pH 7.4). The *C*. *jejuni* suspension was washed with 1X PBS buffer twice and disrupted with a sonicator (Bioruptor Sonication System, Diagenode, USA). After centrifugation at 15,000 rpm for 10 min, the supernatant was used for the assays.

1. **Catalase assay:** Catalase assay was performed according to the manufacturer’s protocol (Amplex Red Hydrogen Peroxide/Peroxidase Assay Kit, Invitrogen). Catalase activities were determined by measuring the intracellular concentration of H_2_O_2_. The supernatant was placed into a 96-well plate and added the working solution (10 mM Amplex Red reagent, 10 U/mL HPR stock solution and reaction buffer). The mixture was incubated at room temperature for 30 min, and fluorescence at ex/em 530/590 nm. H_2_O_2_ concentration was determined by comparing with a standard curve prepared with known H_2_O_2_ concentrations and normalized to protein concentrations determine with a Bradford assay.
2. **SOD assay:** The SOD activity was carried out according to the guidelines of the manufacturer (SOD assay kit, Sigma Aldrich). The supernatant was placed onto a 96 well plate and mixed with the SOD assay mixture (WST working solution, enzyme solution and SOD solution), and the plate was incubated at 37 °C for 20 min. The absorbance at 450 nm was measured with a microplate reader, and the SOD activity was calculated with a SOD standard with normalization to protein concentrations that were determined with a Bradford assay.

### Determination of susceptibility to oxidants

Overnight cultures of *C*. *jejuni* were harvested from MH agar and diluted with fresh MH broth to an optical density 600 of 0.1. The diluted *C*. *jejuni* suspensions were exposed to oxidants, including 2 mM of hydrogen peroxide (H_2_O_2_), 0.2 mM menadione (MND), and 0.1 mM cumene hydroperoxide (CHP), for 1 h. After washing with fresh MH broth twice, the suspension was serially diluted and spread on MH agar.

### Fluorescence microscope analysis

The morphological changes observed with a fluorescence microscope with SYTO9 and propidium iodine staining. *C*. *jejuni* strains were inoculated on MH agar at 42 °C for overnight and harvested with MH broth. The bacterial suspension was serially diluted with fresh MH broth to an OD 600 of 0.07. The *C*. *jejuni* suspension was supplemented with sodium chloride to final concentrations of 1, 2 and 4% and incubated at 42 °C for 5 h with shaking (200 rpm) under microaerobic conditions. After washing with 1X PBS buffer twice, *C*. *jejuni* was fixed with 4% paraformaldehyde for 1 h at room temperature. The cells were centrifuged at 8,000 × *g* for 5 min and washed with 1X PBS buffer twice. After staining with SYTO9 and propidium iodine for 20 min at room temperature and washing twice with 1X PBS buffer, *C*. *jejuni* was observed with a fluorescence microscope (Carl Zeiss, Germany).

### Statistical analysis

Statistical analysis of the data from stress tolerance tests and catalase and SOD assays was performed with two-way ANOVA (GraphPad Prism Ver. 7, GraphPad Software, USA). Chi-square distribution was performed by SPSS ver. 21 (SPSS Inc., IBM, USA).
